# Impact of the First Wave of COVID-19 on the Number of General Anesthesia Cases in 34 Tertiary Hospitals in Japan: A Multicenter Retrospective Study

**DOI:** 10.1155/2021/8144794

**Published:** 2021-08-27

**Authors:** Tomonori Takazawa, Yuki Sugiyama, Yasuhiro Amano, Tetsuhito Hara, Eiki Kanemaru, Takao Kato, Takashi Kawano, Tsukasa Kochiyama, Tatsuya Tsuji, Shigeru Saito

**Affiliations:** ^1^Intensive Care Unit, Gunma University Hospital, Maebashi, Japan; ^2^Department of Anesthesiology and Resuscitology, Shinshu University School of Medicine, Nagano, Japan; ^3^Department of Anesthesiology, Nagoya University Graduate School of Medicine, Nagoya, Japan; ^4^Department of Anesthesiology and Critical Care Medicine, Jichi Medical University, Shimotsuke, Japan; ^5^Department of Anesthesiology, Yokohama City University School of Medicine, Yokohama, Japan; ^6^Department of Anesthesiology, Saitama Medical Center, Saitama Medical University, Saitama, Japan; ^7^Department of Anesthesiology and Intensive Care Medicine, Kochi Medical School, Kochi, Japan; ^8^Department of Anesthesiology and Pain Medicine, Juntendo University Graduate School of Medicine, Tokyo, Japan; ^9^Department of Anesthesiology and Intensive Care Medicine, Graduate School of Medical Sciences, Nagoya City University, Nagoya, Japan

## Abstract

Since the first case of coronavirus disease 2019 (COVID-19) was reported in Japan in January 2020, the COVID-19 pandemic has brought about a significant change in people's lives. Although the COVID-19 pandemic is expected to have had an impact on the work of anesthesiologists, the specific impact has been largely unreported. We hypothesized that the number of general anesthesia (GA) cases has decreased due to the COVID-19 pandemic. To test this hypothesis, we conducted a retrospective survey at 34 facilities in Japan as a part of the Japanese Epidemiologic Study for Perioperative Anaphylaxis. The results showed that the number of GA cases had significantly decreased, particularly in May 2020, under the government's declaration of a state of emergency. The decline in GA caseload had not fully recovered by July 2020. Furthermore, there were regional differences in the decline in the number of GA cases. The impact of the COVID-19 pandemic on the work of anesthesiologists was greater in prefectures where there were more COVID-19 patients and where the state of emergency was declared earlier. Our study suggested a region-dependent decrease in the number of GA cases due to the COVID-19 pandemic.

## 1. Background

Due to the large-scale spread of coronavirus disease 2019 (COVID-19), a state of emergency was declared by the Japanese government in April 2020, which continued until May 25, 2020 [1]. Although the restrictions on human movement were looser in Japan as compared to the lockdowns implemented in European countries and the United States at the same time [2], the restrictions on unnecessary and nonurgent outings had a major impact on the lives of many Japanese people.

At that time, medical facilities were not well prepared to accept COVID-19 patients, and no effective treatment method was established, which caused considerable confusion for healthcare workers [3]. Many patients refrained from going to the hospital because they were afraid of being infected with the virus at the hospital [4]. Besides, medical professionals were overwhelmed by the treatment of COVID-19 patients, and many of them were unable to perform their routine duties [5]. As of May 2021, not only is the COVID-19 pandemic still ongoing but also a third state of emergency has been declared in Japan. As a result, many hospitals have not returned to their prepandemic state. Despite this turmoil, little has been reported about the impact of the COVID-19 pandemic on the work load of Japanese physicians, including anesthesiologists [6]. The purpose of this study was to (1) determine the changes that occurred in the number of general anesthesia (GA) cases during the COVID-19 pandemic; (2) determine whether the change in the number of GA cases was affected by the prevalence of COVID-19 cases; and (3) assess whether there were regional differences in the changes in the number of GA cases.

## 2. Methods

This survey was conducted as a part of the Japanese Epidemiologic Study for Perioperative Anaphylaxis (JESPA), which was registered with the University Hospital Medical Information Network Clinical Trials Registry (ID: 000035350). In this study, we used the data on the number of GA cases collected in the JESPA. The research protocol of the JESPA, including this survey and secondary use of the data, was approved by the ethics committees of the 40 institutions that participated in the study. All patients who opted out of research were excluded from analysis.

The number of GA cases was examined by asking the concerned staff at the participating hospitals to fill out a Microsoft Excel form. GA was defined as anesthesia with loss of consciousness, followed by securing of the airway with endotracheal intubation or supraglottic airway devices. Since the last participating facility joined the survey in July 2020, we analyzed the data for the 13-month period from the start of July 2019 to the end of July 2020. The questionnaire was distributed at the end of July 2020, and responses were accepted until the end of September 2020. The response rate was 85% (34/40).

The number of patients infected with severe acute respiratory syndrome coronavirus 2 (SARS-CoV-2) was obtained from the website of the Ministry of Health, Labour, and Welfare [7]. We also obtained the population estimate data of the Statistics Bureau of the Ministry of Internal Affairs and Communications as of October 1, 2019 [8].

For statistical analysis, various tests, including the Kruskal–Wallis with post hoc Tukey test, Mann–Whitney *U* test, and two-way ANOVA with post hoc Tukey test, were performed depending on the purpose. Pearson's correlation analysis was performed to examine the correlation coefficient. Unless otherwise specified, *P* < 0.05 was considered a significant difference. All statistical analyses were performed using SigmaPlot 14.0 software (Systat Software Inc, San Jose, CA, USA).

## 3. Results

The total number of GA cases at the 34 facilities analyzed in this survey was 162,261. The characteristics of the facilities that responded to the questionnaire were as follows: Twenty facilities were national or public hospitals (59%), while 14 facilities were private hospitals (41%). Twenty-four facilities were university hospitals (71%), and 10 were others (29%). The average number of total beds in each facility was 736 (range: 252–1153). The participating facilities were located in a total of 16 prefectures, as shown in Table 1.

**Figure 1:**
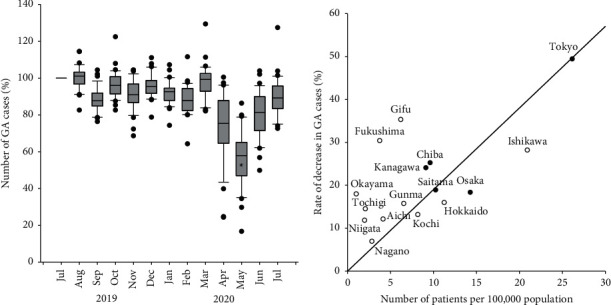
(a) Changes in the number of general anesthesia (GA) cases performed at the 34 facilities from July 2019 to July 2020. The ends of the box define the 25th and 75th percentiles, with the horizontal line in the middle showing the mean and the error bars defining the 10th and 90th percentiles. The dots indicate outliers. ∗, Kruskal–Wallis with the post hoc Tukey test, *P* < 0.001. (b) Relationship between the spread of COVID-19 and the rate of decrease in the number of GA cases in each prefecture. The regression line shows the correlation between them (*R* = 0.66). The prefectures where the state of emergency was issued early and the prefectures where it was issued late are indicated by closed and open circles, respectively.

Figure 1(a) and Supplementary Table 1 display the number of GA cases for each month, normalized versus the July 2019 value. The number of GA cases began to decline in April 2020 and bottomed out in May 2020 (Kruskal–Wallis with the post hoc Tukey test, *P* < 0.001). Although the number of GA cases has been on a recovery trend since then, it had not yet returned to the prepandemic level by July 2020.

Next, we examined whether the change in the number of GA cases was due to the COVID-19 pandemic. The relationship between the number of newly confirmed patients with COVID-19 in each prefecture per 100,000 population and the rate of decrease in the number of GA cases was investigated, and the results are shown in Figure 1(b). The decrease in the number of GA cases was found to correlate with the number of newly confirmed patients with COVID-19 in each prefecture in April 2020; Pearson's correlation coefficient was 0.66. This result suggested that the number of GA cases decreased due to the influence of the COVID-19 pandemic.

**Figure 2:**
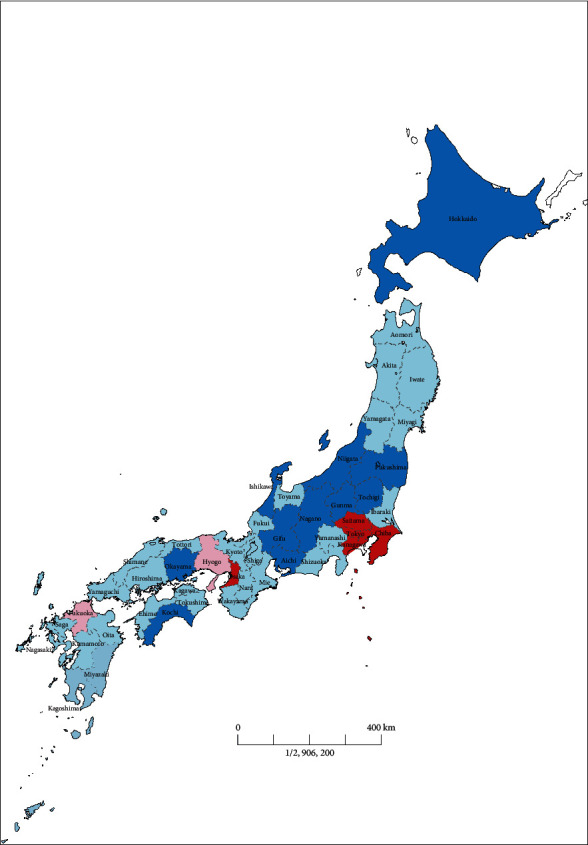
Issuance of the state of emergency by the Japanese government. The early group prefectures, in which the state of emergency was issued on April 7, 2020, are shown in shades of red. The late group prefectures, in which emergency was declared on April 16, 2020, are shown in blue. Although not included in this study, other prefectures where the state of emergency was issued early are shown in light pink, and the prefectures where the state of emergency was issued late are shown in light blue.

Finally, we compared the changes in the number of GA cases in each prefecture to assess regional differences in the impact of the COVID-19 pandemic (Table 1). We divided the 16 prefectures into two groups according to the timing of the issuance of the state of emergency: the early group, in which the state of emergency was issued on April 7, 2020, and the late group, in which emergency was declared on April 16, 2020 (Figure 2). The early group tended to have more newly confirmed COVID-19 patients in April 2020 than the late group (Mann–Whitney *U* test, *P* < 0.05). In prefectures where the state of emergency was issued early, the number of GA cases in April 2020 was lower than that in prefectures where the state of emergency was issued later (two-way ANOVA with the post hoc Tukey test, *P* < 0.05). There was no difference between the two groups in other months. These results suggested regional differences in the work load of Japanese anesthesiologists during the COVID-19 pandemic.

## 4. Discussion

In this study, we found that the number of GA cases in Japan has declined since the spring of 2020 and it is yet to fully recover, probably due to the impact of the COVID-19 pandemic. Furthermore, such changes vary depending on the prefecture.

During the period we investigated, the decrease in the number of GA cases in April and May 2020 was particularly noticeable (Figure 1 and Table 1). These results are consistent with the results of previous studies conducted in Japan using Twitter Polls [9]. Given the correlation between the rate of decrease in GA cases and the number of patients with COVID-19, it is reasonable to interpret that the number of GA cases decreased due to the COVID-19 pandemic. Furthermore, April 2020, when the number of GA cases began to decrease, coincides with the time when the Japanese government issued a state of emergency. In other words, the government decision had a great influence on the work of anesthesiologists. This was supported by the fact that the degree of decrease in the number of GA cases differed depending on when the state of emergency was issued. Since the state of emergency was issued early in areas where SARS-CoV-2 infection was severe, one could paraphrase that the degree of spread of infection had a direct impact on the work load of anesthesiologists. However, it is important to note that the issuance of a state of emergency is determined not only by the number of COVID-19 patients but also by other factors, including the pressure on medical resources.

This study has certain limitations, as described below. First, due to the small number of facilities surveyed, the results of this study do not necessarily reflect the overall situation in Japan. Indeed, only one facility per prefecture participated in the survey in many prefectures. For example, the COVID-19 status might vary from region to region even within the same prefecture. In such prefectures, the potential risk of a large deviation from the actual situation cannot be excluded. Furthermore, it is unclear how the fact that most of the participating institutions in this study were university hospitals (71%) might have affected the study results. A survey with more facilities will need to be conducted in the future. Second, the number of GA cases was normalized versus that in July 2019, when the last facility joined the study. Although we obtained data from some institutions for the number of GA cases from February to June 2019, data from all institutions were available only after July 2019. Since the number of GA cases is expected to have a seasonal effect, it would be ideal if the number of GA cases in each month could be displayed year on year. Slightly more holidays in May compared to other months might also have affected the number of GA cases. Third, since this study focused on the first wave of the COVID-19 pandemic, it is unclear whether the results of this study can be applied to subsequent waves.

## 5. Conclusions

Our study suggested that the COVID-19 pandemic reduced the number of GA cases. Besides, there was a clear regional difference in the degree of decrease. This suggests that anesthesiologists might need to change the way they work when there is further deterioration in the COVID-19 pandemic situation.

## Figures and Tables

**Table 1:** Comparison of the number of general anesthesia cases in each prefecture. The “number of patients with COVID-19” reflects the number of patients per 100,000 population in each prefecture as of April 2020.

State of emergency	Prefectures	Number of facilities	Number of patients with COVID-19	Number of GA cases (%)						
				Jan	Feb	Mar	Apr	May	Jun	Jul
Early issued	Tokyo	9	26.1	94.1	88.7	97.8	50.6	37.8	67.3	84.1
	Osaka	1	25.4	92.4	89.7	99.8	81.6	65.5	90.2	99.3
	Saitama	4	10.2	93.9	90.5	97.1	81.1	59.6	82.0	86.5
	Chiba	1	9.6	87.5	84.4	91.1	74.8	59.2	86.5	94.5
	Kanagawa	1	9.1	83.9	80.0	91.4	75.9	58.2	79.4	93.5
	Average		16.1	90.4	86.7	95.5	72.8	56.1	81.1	91.6

Late issued	Ishikawa	1	20.9	93.9	95.2	103.2	71.8	47.6	71.3	75.0
	Hokkaido	1	11.2	94.5	90.8	82.4	84.0	47.4	70.2	88.2
	Kochi	1	8.2	94.3	94.3	92.9	86.8	77.7	94.6	100.7
	Gunma	7	6.5	90.7	86.4	102.7	84.2	67.5	89.2	96.4
	Gifu	1	6.2	88.2	80.2	93.0	64.7	57.0	85.3	81.3
	Aichi	2	4.1	92.0	89.8	99.3	87.9	59.8	76.7	91.5
	Fukushima	1	3.7	91.6	88.2	98.4	69.6	52.4	72.9	87.2
	Nagano	1	2.8	99.0	97.7	111.3	93.0	79.9	102.1	103.9
	Tochigi	1	2.1	90.1	85.3	100.2	85.5	78.4	97.2	96.5
	Niigata	1	2.0	87.9	82.3	88.8	88.1	65.1	83.2	86.1
	Okayama	1	1.0	86.1	83.3	98.5	82.0	57.6	76.8	85.7
	Average		6.3^∗^	91.7	88.5	97.3	81.6^†^	62.8	83.6	90.2

The “number of GA cases” reflects the number of GA cases in each prefecture normalized versus the number in July 2019. The data after January 2020, when the first case of COVID-19 was reported in Japan, are shown. Values with significant differences between the two groups are shown in bold. The symbols indicate significant differences between groups. ^∗,†^Early vs. late group (*P* < 0.05).

## Data Availability

The datasets used during the current study are available from the corresponding author on reasonable request.

## Supplementary Materials

Supplementary Materials Supplementary Table 1. Changes in the number of general anesthesia cases performed at 34 facilities in Japan from July 2019 to July 2020. The numbers in the first line show the median ratio of the number of general anesthesia cases in each month to the number of general anesthesia cases in July 2019 at each institution. The numbers in the second and third lines show the 25th and 75th percentile values, respectively, of the data shown in the first line.
