# Supplementary material for: Impact of the First Wave of COVID-19 on the Number of General Anesthesia Cases in 34 Tertiary Hospitals in Japan: A Multicenter Retrospective Study
Source: Anesthesiol Res Pract. 2021 Aug 27;2021:8144794. doi: 10.1155/2021/8144794 (PMC8426062; doi:10.1155/2021/8144794)
Supplement: Supplementary Materials — Supplementary Table 1. Changes in the number of general anesthesia cases performed at 34 facilities in Japan from July 2019 to July 2020. The numbers in the first line show the median ratio of the number of general anesthesia cases in each month to the number of general anesthesia cases in July 2019 at each institution. The numbers in the second and third lines show the 25th and 75th percentile values, respectively, of the data shown in the first line. [file 8144794.f1.docx]

**Supplementary Table 1**

Changes in the number of general anesthesia cases performed at 34 facilities in Japan from July 2019 to July 2020

| Month | 2019 | | | | | | 2020 | | | | | | |
| --- | --- | --- | --- | --- | --- | --- | --- | --- | --- | --- | --- | --- | --- |
|  | Jul | Aug | Sep | Oct | Nov | Dec | Jan | Feb | Mar | Apr | May | Jun | Jul |
| Median | 100.0 | 101.1 | 87.7 | 96.0 | 90.9 | 95.5 | 92.7 | 87.9 | 99.3 | 75.3 | 57.9 | 81.4 | 89.3 |
| 25% | 100.0 | 96.7 | 84.8 | 91.3 | 86.7 | 91.7 | 87.8 | 82.3 | 92.8 | 64.5 | 47.0 | 71.5 | 83.4 |
| 75% | 100.0 | 103.3 | 92.0 | 100.8 | 96.8 | 98.7 | 94.6 | 94.4 | 102.5 | 87.8 | 65.2 | 90.0 | 95.8 |

The numbers in the first line show the median ratio of the number of general anesthesia cases in each month to the number of general anesthesia cases in July 2019 at each institution. The numbers in the second and third lines show the 25th and 75th percentile values, respectively, of the data shown in the first line.
